# Symptomatic Post-anastomotic Blind Ileal Pouch

**DOI:** 10.7759/cureus.117974

**Published:** 2026-10-15

**Authors:** Luis F Flores, Tatiana Fernandez Trokhimtchouk, Monica Orejuela, Elena Leon

**Affiliations:** 1 General Surgery, Nuevo Hospital Inglés, Quito, ECU; 2 General Surgery, Universidad de Las Américas, Quito, ECU; 3 General Surgery, Hospital General del Sur de Quito, Instituto Ecuatoriano de Seguridad Social, Quito, ECU; 4 General Surgery, Hospital de Especialidades Carlos Andrade Marín, Quito, ECU

**Keywords:** acute abdomen, blind loop syndrome, blind pouch syndrome, chronic diarrhea, ileocecal anastomosis, postsurgical complication, side-to-side anastomosis, small bowel resection

## Abstract

Postsurgical blind pouch syndrome is an uncommon late complication of intestinal reconstruction caused by stasis within a poorly emptying bowel segment. Its manifestations range from chronic diarrhea and abdominal discomfort to bleeding, obstruction, inflammation, and perforation, while diagnosis may be difficult when the previous operative anatomy is unknown. We report the case of a 48-year-old man who presented with severe right-sided abdominal pain, distension, and peritoneal irritation three years after emergency bowel resection and an undocumented intestinal anastomosis following a motor vehicle collision. Since the previous operation, he had experienced frequent bowel movements and fecal urgency that substantially impaired his quality of life. Initial computed tomography and diagnostic laparoscopy failed to identify a definitive cause of his symptoms, and extensive adhesions prevented complete characterization of the reconstruction. Persistent severe pain, fever, and marked elevation of inflammatory markers prompted repeat exploration five days later. Laparotomy demonstrated a patent side-to-side ileocecal anastomosis associated with a markedly dilated and thickened blind ileal pouch and a separate atrophic terminal ileal remnant. The blind pouch was resected. Histopathological examination revealed superficial mucosal ulceration, muscular hypertrophy, edema, hemorrhage, focal liquefactive necrosis, and acute-on-chronic transmural inflammation without malignancy. Recovery was uneventful. At three-month follow-up, the patient remained asymptomatic and medication-free, with normal bowel function and complete resolution of fecal urgency and abdominal pain. This case highlights that a postsurgical blind ileal pouch may cause severe acute inflammation even without gross perforation and should be considered in patients with unexplained abdominal symptoms after intestinal reconstruction.

## Introduction

Blind loop syndrome traditionally describes the clinical consequences of intestinal stasis and bacterial overgrowth, including abdominal pain, diarrhea, weight loss, anemia, vitamin deficiencies, and malabsorption [1]. Within this broader entity, blind pouch syndrome refers more specifically to the mechanical and clinical consequences of a non-emptying intestinal cul-de-sac created by a side-to-side or end-to-side anastomosis [2,3]. Abnormal peristalsis may direct intestinal contents toward the closed end, promoting stasis, bacterial overgrowth, progressive pouch dilatation, and chronic inflammatory changes.

Clinical manifestations are variable and may develop years after the original operation. Patients may present with nonspecific abdominal pain, diarrhea, malabsorption, anemia, or weight loss. Less commonly, progressive dilatation and inflammation may result in enterolith or fecalith formation, intestinal obstruction, mucosal ulceration, gastrointestinal bleeding, or perforation [4-7]. Computed tomography (CT) may demonstrate a focally dilated bowel segment adjacent to an anastomotic staple line; however, the abnormality can be mistaken for a diverticulum, abscess, or obstructed bowel loop, particularly when the previous operative anatomy is unknown [4,8].

The incidence and natural history of post-anastomotic blind pouches remain poorly defined. Available evidence consists predominantly of historical descriptions, small radiological series, and isolated case reports. Most studies of intestinal anastomoses focus on short-term outcomes rather than delayed anatomical complications [4,6]. The purpose of this report is to describe a diagnostically challenging symptomatic blind ileal pouch presenting as acute abdominal pain three years after an undocumented intestinal reconstruction. Although no gross perforation was identified, histopathological examination demonstrated ulcerative, inflammatory, and necrotic changes, and resection resulted in sustained resolution of both the acute presentation and the patient’s longstanding bowel dysfunction.

## Case presentation

A 48-year-old man presented with a six-hour history of sudden-onset, severe colicky abdominal pain involving the right flank and right lower quadrant, associated with abdominal distension and nausea without vomiting. His surgical history included exploratory laparotomy, resection of approximately 1 m of bowel, and intestinal anastomosis following a motor vehicle collision three years earlier. The original operative records were unavailable; therefore, information regarding the extent of resection and the configuration of the reconstruction was based on the patient’s account.

Since the previous operation, the patient had experienced frequent bowel movements and fecal urgency that substantially impaired his quality of life. His symptoms had not improved with dietary modification or probiotics. He also reported undergoing a previous colonoscopy without relevant abnormalities.

On initial examination, he was alert, afebrile, and hemodynamically stable but appeared distressed by pain. The abdomen was distended and diffusely tender, with signs of peritoneal irritation and decreased bowel sounds. Laboratory investigations demonstrated a leukocyte count of 12.15 × 10³/µL with 82.3% neutrophils, hemoglobin of 14.8 g/dL, creatinine of 1.23 mg/dL, and serum lactate of 3.98 mmol/L. An unenhanced CT scan obtained at the referring institution was interpreted as suggestive of pneumoperitoneum and inflammatory changes involving the terminal ileum. Because neither the original images nor a formal radiology report was available, the imaging basis for this interpretation could not be independently assessed.

Contrast-enhanced abdominal CT performed at the receiving institution before surgery showed no free intraperitoneal fluid or evident disruption of the previous intestinal anastomosis. Retrospective review of the multiplanar images demonstrated a staple line corresponding to a side-to-side ileocecal anastomosis and the contours of an adjacent blind ileal pouch (Figure 1). A short, atrophic terminal ileal segment extending toward the preserved ileocecal valve and the appendix were also identifiable (Figure 2). However, the significance of this altered anatomy was not recognized at the time.

**Figure 1:**
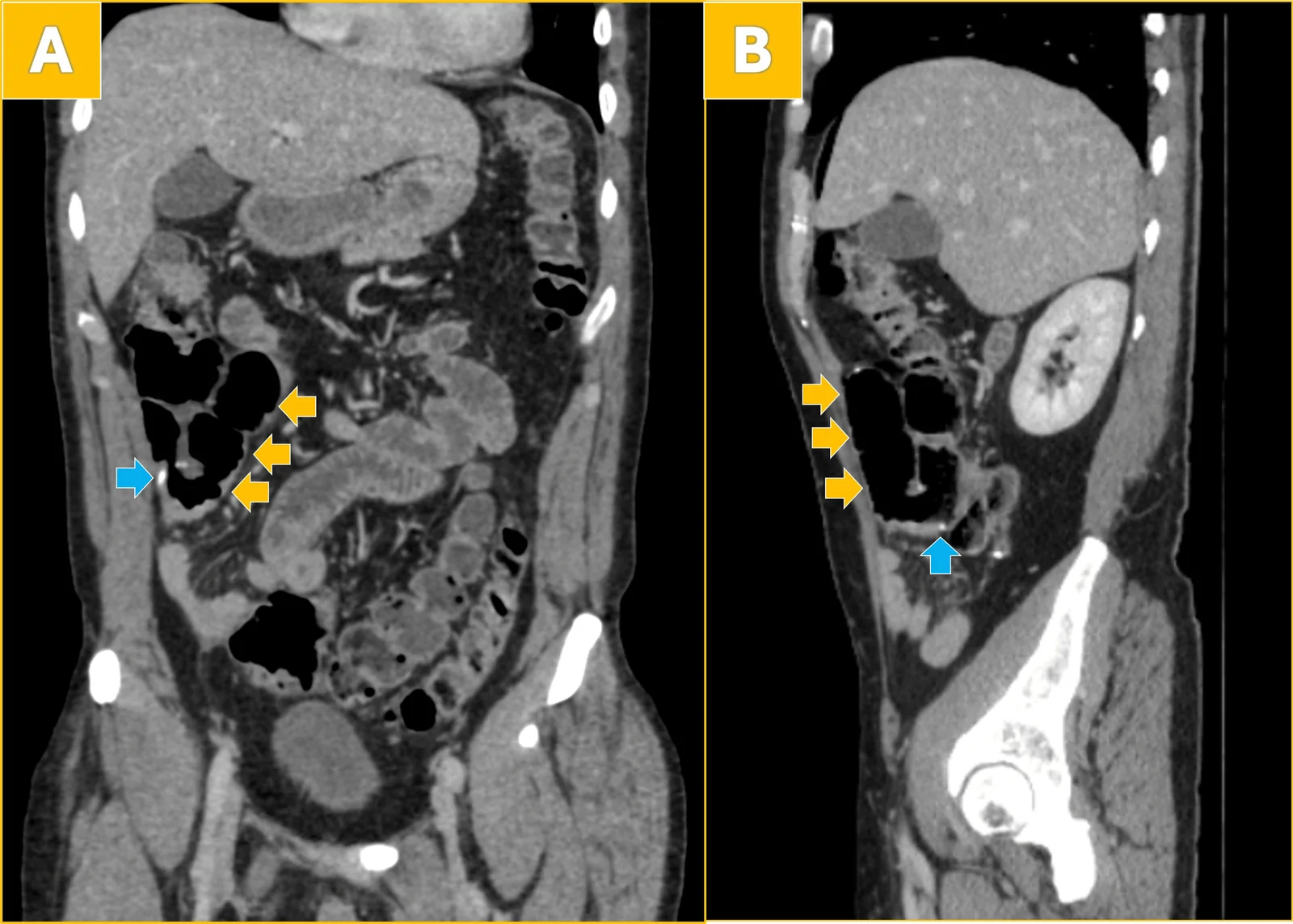
Preoperative CT demonstrating the post-anastomotic blind ileal pouch (A) Coronal and (B) sagittal contrast-enhanced CT reconstructions obtained before the initial laparoscopy. The blue arrow indicates the staple line of the side-to-side ileocecal anastomosis, while the yellow arrows delineate the margins of the blind ileal pouch. CT: computed tomography

**Figure 2:**
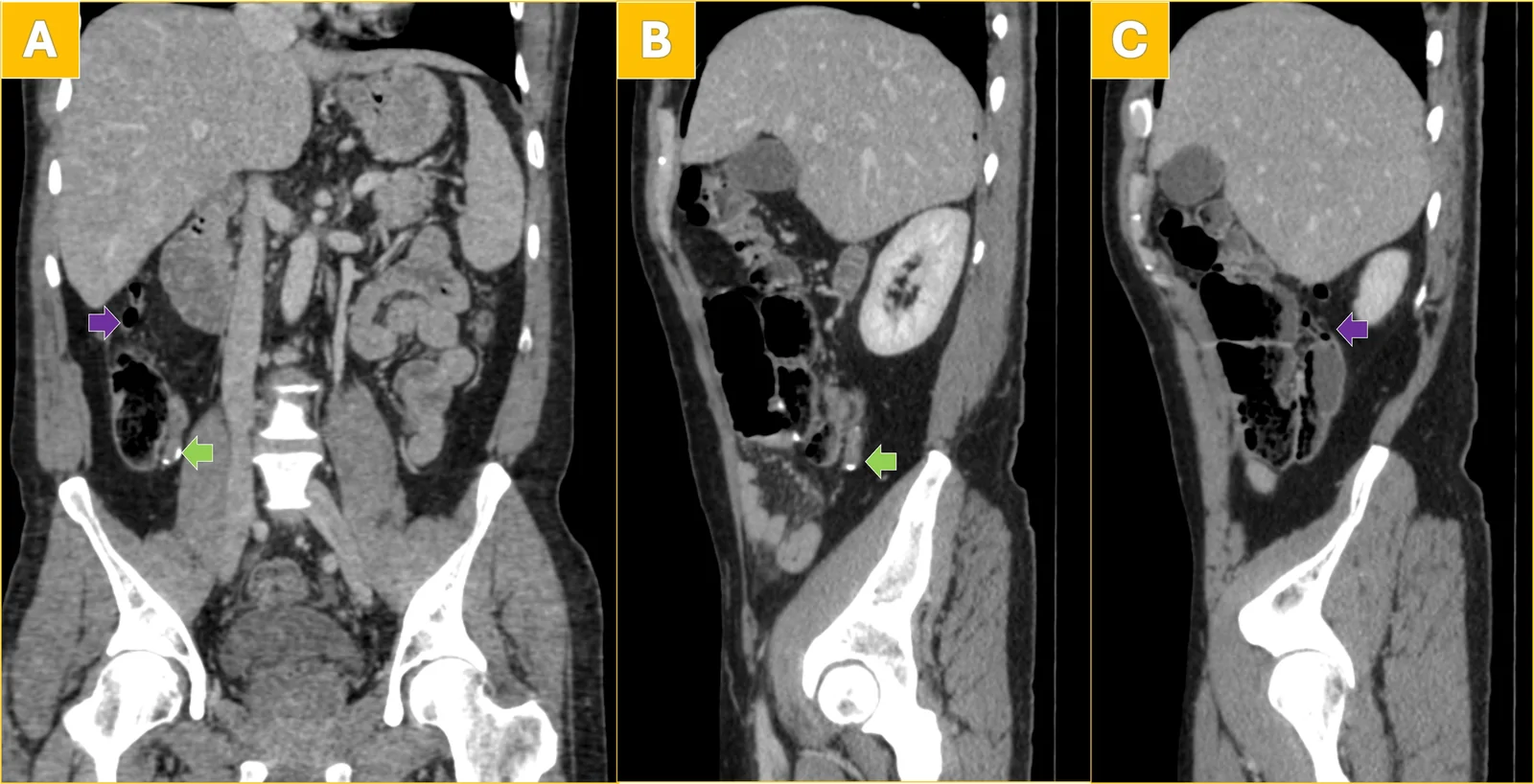
Preoperative CT demonstrating the excluded terminal ileal segment and preserved appendix (A) Coronal and (B-C) sagittal contrast-enhanced CT reconstructions obtained before the initial laparoscopy. The green arrow indicates the short, atrophic terminal ileal segment extending toward the preserved ileocecal valve, and the purple arrow indicates the appendix. CT: computed tomography

Because of the severity of the pain, signs of peritoneal irritation, elevated lactate, and concern for an intra-abdominal condition requiring surgery, diagnostic laparoscopy was performed. Severe adhesions involving the omentum and small-bowel loops along the previous midline laparotomy incision were encountered. The appendix was erythematous and located in a subhepatic position because of cephalad rotation of the cecum. Two adhesive bands were identified: one between the small bowel and sigmoid colon and another between two small-bowel loops. The region of the previous intestinal operation was identified; however, the configuration of the reconstruction could not be completely defined laparoscopically because of the extensive adhesions. No gastrointestinal perforation, anastomotic leak, or other definitive cause of the acute presentation was identified. Laparoscopic adhesiolysis and appendectomy were performed without complications.

The patient initially recovered satisfactorily, tolerated oral intake, passed flatus, and was discharged on the first postoperative day. Approximately 12 hours after discharge, following food intake, he developed recurrent severe colicky abdominal pain, abdominal distension, and inability to pass flatus, prompting his return to the emergency department. At readmission, the abdomen remained distended and diffusely tender, although there was no hemodynamic deterioration. Laboratory testing showed no leukocytosis, preserved renal function, and persistent hyperlactatemia. His symptoms initially improved with intravenous hydration, analgesia, and prokinetic therapy, and he was admitted with a presumed postoperative ileus.

Although intestinal transit subsequently returned, the abdominal pain persisted. Colonoscopy was attempted during the first day after readmission but was discontinued because of inadequate bowel preparation. Upper gastrointestinal endoscopy with gastric and duodenal biopsies was subsequently performed. Histopathological examination demonstrated mild inactive chronic gastritis without Helicobacter pylori, atrophy, or intestinal metaplasia. Duodenal biopsies showed mild inactive chronic duodenitis with mild villous atrophy and no evidence of malignancy. These findings did not adequately explain the severity of the clinical presentation.

Following the endoscopic evaluations, the patient developed a temperature of 38.1°C and continued to experience recurrent abdominal pain. Laboratory testing demonstrated a C-reactive protein concentration of 301 mg/L and a procalcitonin concentration of 0.31 ng/mL, with a normal leukocyte count. After administration of oral and rectal contrast, he developed another episode of sudden, severe right lower quadrant pain. Repeat contrast-enhanced abdominal CT, performed after the initial laparoscopy and before definitive re-exploration, was not prospectively interpreted as showing a cause for the persistent symptoms. However, retrospective review showed marked gaseous distension of the blind ileal pouch, with the staple line visible along its border (Figure 3).

**Figure 3:**
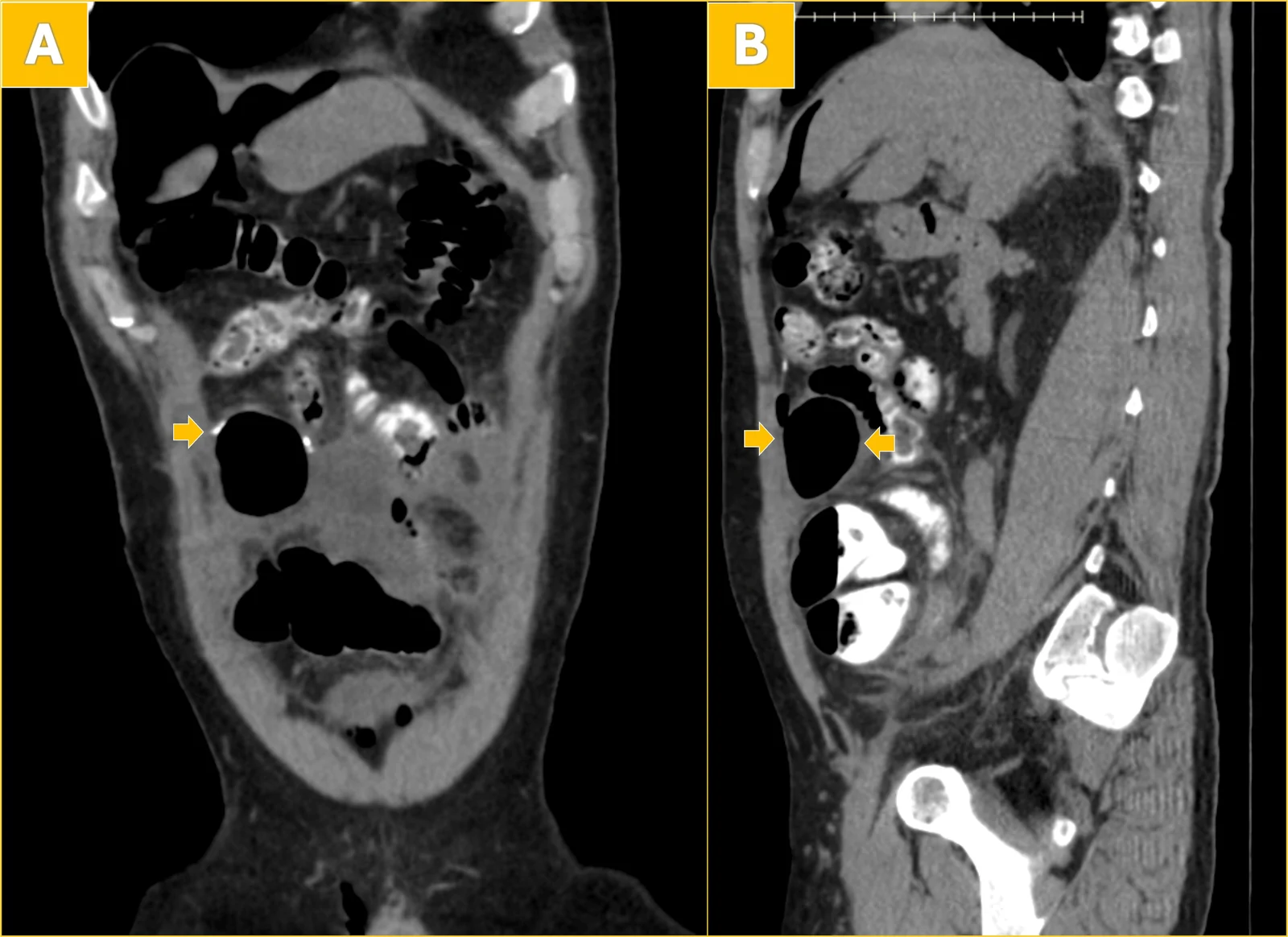
Post-laparoscopy CT demonstrating distension of the blind ileal pouch (A) Coronal and (B) sagittal CT reconstructions obtained after enteric contrast administration, following the initial laparoscopy and before definitive re-exploration. The blind ileal pouch is markedly distended with gas. Yellow arrows indicate the staple line along its border; enteric contrast is visible within adjacent bowel loops. CT: computed tomography

Despite conservative treatment and opioid analgesia, the patient continued to experience severe pain. Given the absence of a plausible nonsurgical diagnosis and the persistence and severity of his symptoms, repeat surgical exploration was undertaken five days after the initial operation.

The procedure was initiated laparoscopically but converted to laparotomy because of dense adhesions and difficulty defining the previous reconstruction. Approximately 70 mL of inflammatory fluid was found within the peritoneal cavity. Severe adhesions involved approximately 30 cm of small bowel and produced multiple angulations. Fibrinous tissue was present in the right paracolic gutter at the site of the previous adhesiolysis, without evidence of intestinal leakage.

Complete exploration demonstrated a patent side-to-side anastomosis between the ileum and cecum. The reconstruction had left a markedly dilated and thickened blind ileal pouch measuring approximately 15 cm (Figure 4). A separate, short segment of excluded terminal ileum extended toward the preserved ileocecal valve. This segment was atrophic but not dilated and showed no other gross pathological changes; it was therefore left in situ to avoid unnecessary additional dissection. The pre-existing ileocecal reconstruction and the resulting blind pouch are schematically illustrated in Figure 5. The appendix was removed during the preceding operation, and its stump remained intact. No gross ulceration, perforation, anastomotic dehiscence, or gross intestinal necrosis was observed. The remaining small bowel measured approximately 230 cm from the ligament of Treitz to the ileocecal anastomosis. The dilated blind ileal pouch was resected, and extensive adhesiolysis was completed.

**Figure 4:**
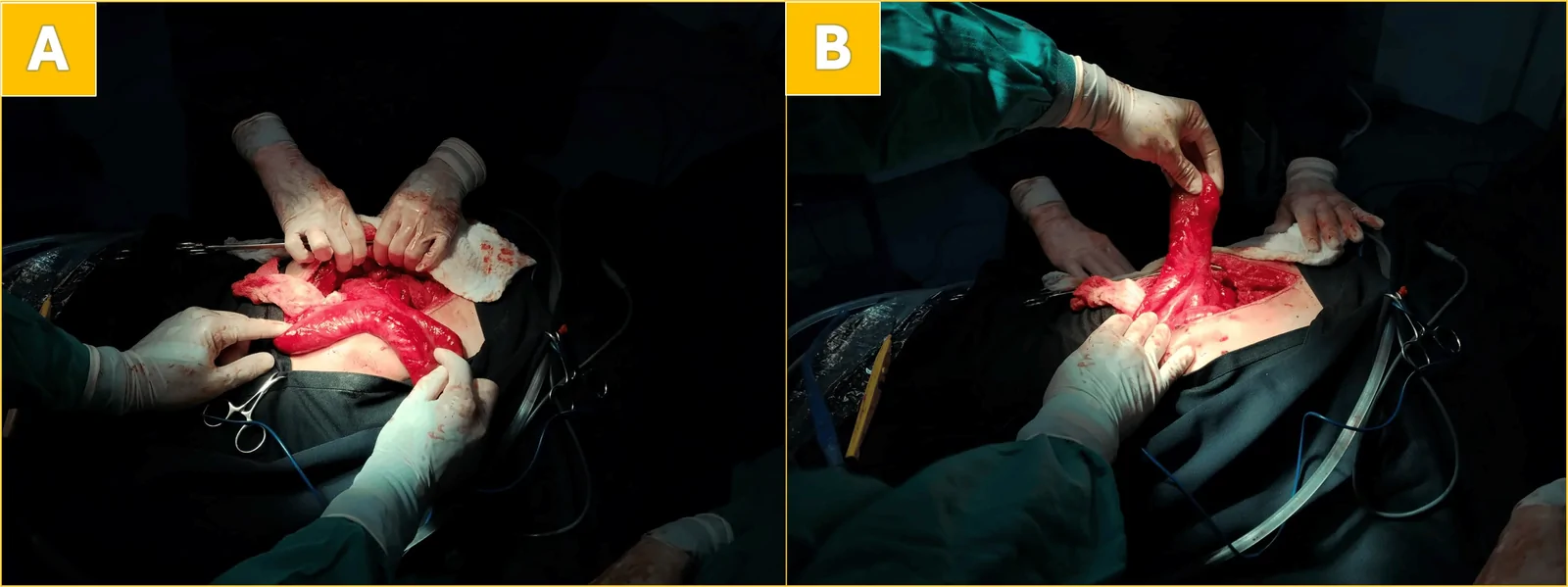
Intraoperative appearance of the post-anastomotic blind ileal pouch (A) The markedly dilated and thickened blind ileal pouch is shown in situ during open exploration. (B) Elevation of the pouch demonstrates its elongated, saccular configuration before resection.

**Figure 5:**
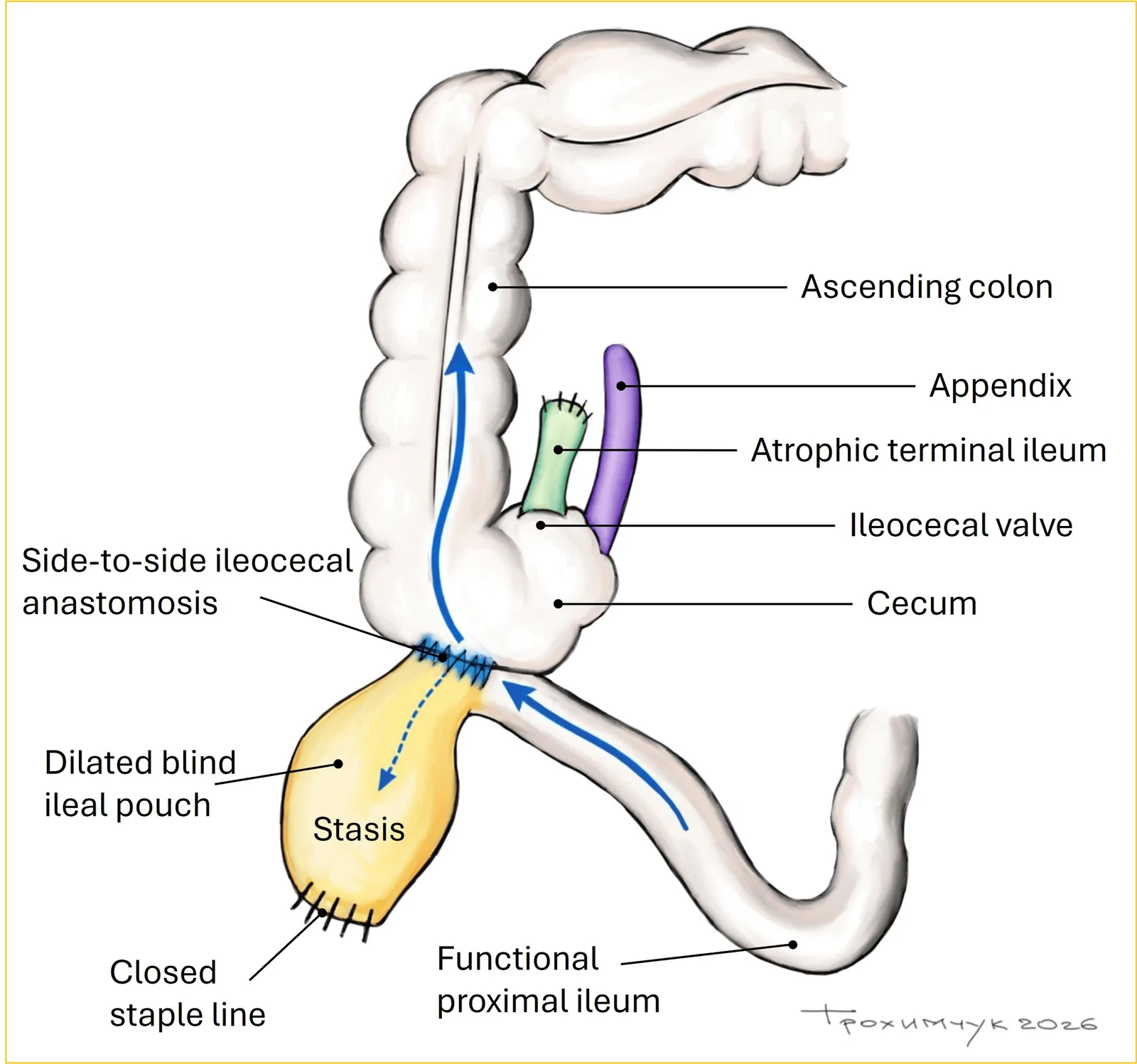
Schematic representation of the pre-existing ileocecal reconstruction The functional proximal ileum formed a side-to-side anastomosis with the cecum and continued beyond it into a dependent, markedly dilated blind-ending ileal pouch closed by a staple line. A short excluded terminal ileal segment extended toward the preserved ileocecal valve and had become atrophic. Solid blue arrows indicate the presumed physiological direction of intestinal transit, whereas the dashed arrow indicates stasis within the blind pouch. The appendix is depicted to reconstruct the anatomy before the initial laparoscopy and appendectomy. Image Credit: The illustration was manually drawn by T. Trokhimtchouk using Sketchbook Pro (Sketchbook, Inc., San Rafael, CA, USA) and Krita (Krita Foundation, Deventer, the Netherlands) based on the imaging and operative findings.

Gross pathological examination showed a saccular segment of small bowel measuring 6.5 × 5 × 3 cm, with a dull violaceous serosal surface, scant fibrinous material, adherent blood clots, and mural thickening of up to 0.7 cm. Microscopic examination demonstrated superficial mucosal ulceration, muscular hypertrophy, marked edema, hemorrhage, neovascularization, vascular ectasia and congestion, mucin extravasation, focal liquefactive necrosis, and acute-on-chronic transmural inflammation. The surgical margin was viable, and no malignancy was identified. The pathological diagnosis was necrosis and acute inflammation associated with a blind ileal pouch.

The postoperative course was uneventful. Oral intake was progressively resumed, intestinal transit returned, and pain was adequately controlled with oral analgesia. The patient was discharged on the fourth postoperative day in stable condition. At follow-up one week later, he reported normalization of bowel function. At three months, he remained asymptomatic and medication-free, with normal bowel frequency, resolution of fecal urgency and abdominal pain, and complete return to his usual daily and occupational activities.

## Discussion

Postsurgical blind pouch syndrome is an uncommon and probably underrecognized late complication of intestinal reconstruction. More specifically, it describes the clinical consequences of a poorly emptying intestinal cul-de-sac created by a side-to-side anastomosis and should be distinguished from the broader blind loop syndrome, which may arise from several anatomical or motility disorders [3,9,10]. Abnormal propulsion into this cul-de-sac may promote progressive dilatation, luminal stasis, and bacterial overgrowth, which may produce diarrhea, abdominal pain, malabsorption, anemia, and nutritional deficiencies. Continued distension can also cause mural inflammation, ulceration, bleeding, enterolith formation, obstruction, and, rarely, perforation [1-4,9]. Its true incidence remains unknown because most evidence consists of isolated reports or small case series, and complications may arise several years after the original operation.

The anatomical configuration identified in this patient differed from the more frequently reported blind ileal stump following right hemicolectomy. The cecum, appendix, ileocecal valve, and a short atrophic terminal ileal segment remained, while the functional ileum was anastomosed side-to-side to the cecum. The ileum continued beyond the anastomosis into a long, dependent blind pouch. This arrangement created a potential reservoir into which intestinal contents could enter but from which effective emptying was impaired. Similar configurations have been described as creating an antiperistaltic or functionally excluded segment in which peristaltic activity directs intestinal contents toward the closed end rather than along the physiological route [2,10,11].

The clinical manifestations of a blind pouch vary according to the degree of stasis and the complications that develop. Chronic diarrhea and abdominal discomfort are traditionally attributed to stasis and bacterial overgrowth, with deconjugation of bile salts and impaired nutrient absorption [1,3,9]. However, bacterial overgrowth was not formally demonstrated in this patient; therefore, his longstanding increased bowel movement frequency and fecal urgency cannot be conclusively attributed to this mechanism. Nevertheless, their onset after the previous reconstruction, failure to respond to dietary modification and probiotics, and complete resolution following resection of the blind pouch support a clinically meaningful association. Similar improvement in pain and bowel function after treatment of an ileocolic blind loop has been described previously [2].

The acute presentation was particularly difficult to diagnose. Most recently reported acute complications have involved perforation of a progressively enlarged blind ileal segment, often years after a side-to-side ileocolic anastomosis [4-6]. By contrast, this patient had no perforation, anastomotic dehiscence, or gross intestinal necrosis. Histopathological examination nevertheless demonstrated superficial mucosal ulceration, muscular hypertrophy, marked edema, hemorrhage, vascular congestion, focal liquefactive necrosis, and acute-on-chronic transmural inflammation. These findings provide a pathological correlate for the severe pain and systemic inflammatory response despite the absence of gross perforation. Muscular hypertrophy and chronic inflammatory changes further suggest that the process evolved over time rather than representing an exclusively postoperative event.

CT is considered the most useful imaging modality for recognizing a postsurgical blind pouch. The characteristic finding is a focally dilated, pouch-like small-bowel segment near an anastomosis or surgical staples, sometimes accompanied by mural thickening, mucosal hyperenhancement, surrounding fat stranding, enteroliths, free fluid, or extraluminal gas when perforation has occurred [4,6,8]. Nevertheless, these findings are nonspecific, and a blind pouch may be mistaken for a diverticulum, abscess, or obstructed bowel segment. In the present case, the altered postoperative anatomy was not recognized prospectively, and the lack of previous operative documentation further limited interpretation. Retrospective review, particularly of multiplanar images obtained after luminal contrast administration, demonstrated the markedly distended gas-filled pouch adjacent to the staple line. The episode of severe pain after enteral contrast administration raises the possibility that increased intraluminal volume transiently aggravated distension within the poorly emptying pouch. Although causality cannot be established, this temporal association suggests that, when abnormal postoperative anatomy or a closed-loop process is suspected, the diagnostic benefit of enteral contrast should be weighed against the possibility of worsening distension.

The first operation also illustrates the limitations of laparoscopy in patients with dense postoperative adhesions and complex, undocumented reconstruction. Although the previously operated region was identified, its exact configuration could not be safely characterized, and no perforation or leak was found. The initial adhesiolysis may have treated a potential adhesive component, but it did not address the blind pouch. Persistent severe pain, fever, markedly elevated C-reactive protein, and the absence of a convincing alternative diagnosis supported the decision to perform repeat exploration. Conversion to laparotomy permitted complete definition of the reconstruction and identification of the diseased pouch.

Treatment depends on the clinical manifestations. Antibiotics and nutritional correction may temporarily improve symptoms associated with bacterial overgrowth, but they do not correct the underlying anatomical reservoir, and recurrence may occur [2]. Complicated blind pouches that cause obstruction, bleeding, severe inflammation, necrosis, or perforation generally require surgery. Reported options include resection of the blind segment alone or revision of the reconstruction, usually to eliminate the poorly emptying cul-de-sac [4-7,9,10]. Because the ileocecal anastomosis in this patient was patent and viable, limited resection of the diseased blind pouch avoided construction of a new anastomosis while removing the suspected source of inflammation and stasis.

This case emphasizes the importance of minimizing the length of any blind intestinal stump created during side-to-side reconstruction and documenting unusual postoperative anatomy. It also expands the clinical spectrum of ileocolic blind pouch complications: severe acute abdominal pain may arise from acute-on-chronic transmural inflammation and focal necrosis even in the absence of gross perforation. In patients with unexplained chronic bowel symptoms or an acute abdomen after intestinal reconstruction, careful review of the anastomotic configuration and adjacent excluded segments should be incorporated into both radiological interpretation and surgical exploration. Normalization of bowel function and complete pain resolution after pouch resection support the clinical relevance of the abnormal anatomy in this patient. Nevertheless, this report is limited by the absence of documentation from the original operation, the lack of objective testing for bacterial overgrowth or malabsorption, and the relatively short follow-up period; therefore, the causal relationship between the pouch and the previous chronic bowel symptoms should be interpreted cautiously.

## Conclusions

Postsurgical blind ileal pouches are uncommon but clinically important late complications of intestinal reconstruction. They should be considered in patients with unexplained chronic gastrointestinal symptoms or acute abdominal pain after a side-to-side anastomosis, particularly when the original operative anatomy is unknown. Careful multiplanar CT review may identify a dilated bowel segment adjacent to the anastomotic staple line, although dense adhesions and complex reconstruction may ultimately require complete surgical exploration. In this case, resection of the blind pouch resolved both the acute abdominal pain and the longstanding bowel dysfunction. The pathological findings demonstrate that severe acute-on-chronic inflammation and focal necrosis may occur even without gross perforation. When constructing a side-to-side anastomosis, the residual blind segment should be kept as short as technically possible, although this practice does not eliminate the risk of subsequent dilatation and stasis.
